# Analysis of Heat Dissipation and Reliability in Information Erasure: A Gaussian Mixture Approach

**DOI:** 10.3390/e20100749

**Published:** 2018-09-30

**Authors:** Saurav Talukdar, Shreyas Bhaban, James Melbourne, Murti Salapaka

**Affiliations:** 1 Department of Mechanical Engineering, University of Minnesota-Twin Cities, Minneapolis, MN 55455, USA; 2 Department of Electrical and Computer Engineering, University of Minnesota-Twin Cities, Minneapolis, MN 55455, USA

**Keywords:** information erasure, generalized Landauer’s principle, thermodynamics of information, reliability of a bit

## Abstract

This article analyzes the effect of imperfections in physically realizable memory. Motivated by the realization of a bit as a Brownian particle within a double well potential, we investigate the energetics of an erasure protocol under a Gaussian mixture model. We obtain sharp quantitative entropy bounds that not only give rigorous justification for heuristics utilized in prior works, but also provide a guide toward the minimal scale at which an erasure protocol can be performed. We also compare the results obtained with the mean escape times from double wells to ensure reliability of the memory. The article quantifies the effect of overlap of two Gaussians on the the loss of interpretability of the state of a one bit memory, the required heat dissipated in partially successful erasures and reliability of information stored in a memory bit.

## 1. Introduction

Over the last four decades, the semiconductor industry has made significant headway in improving the performance of complementary metal oxide semiconductor field effect transistor (CMOS-FET) devices, while consistently reducing their size. It has enabled tremendous improvements in the capabilities of personal computers, laptops and smart phones—to the point where a modern smart phone is several hundred times faster than the computational abilities of devices that guided the NASA’s travel to the moon in 1969. A crucial enabler is the increase in energy efficiency of computing devices without compromising performance.

Recent research is focused toward investigating fundamental limits on how efficient a device can be or how small its physical dimensions can get. Subsequent fundamental contributions [1,2,3] demonstrated a link between the fields of Thermodynamics and Information Theory, leading to an important understanding of the limits on the consumption of energy in performing bit level operations.

**Figure 1:**
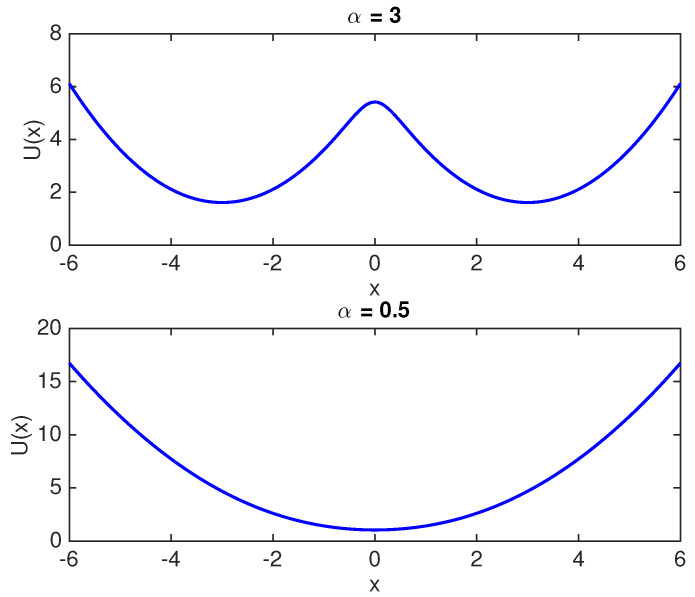
A symmetric double well potential, U(x), where the location of the particle in the left and right well designate the state zero and one respectively of a single bit memory. Here, *E* denotes the barrier height.

A prevalent model of a single bit memory is a Brownian particle in a symmetric double well potential in a heat bath at constant temperature *T*, with the two identical wells separated by a barrier as shown in Figure 1. The presence of the particle in either well denotes one of the possible two states of a single bit memory. In this article, we designate the particle’s presence in the left well and right well as state zero and one of the memory, respectively. The presence of an energy barrier of sufficient height ($\gg k_{B}T$, $k_{B}$ is the Boltzmann constant) ensures that information is retained for a long duration, as is desired from a memory bit. A shorter barrier height ($\approx k_{B}T$) will result in a higher probability of the particle moving from one well to another due to thermal fluctuations, which is undesirable as it renders the information contained in the memory bit unreliable. Erasure of a bit of information is reset to zero operation, where we consider the erasure process successful if the particle is in the left well at the termination of the erasure process irrespective of the initial state of the memory.

Landauer’s principle [1] asserts that erasure of a bit of information, provided both states of a single bit memory are identical, is accompanied by an average heat dissipation of at least $k_{B}T\ln2$. Landauer argued that erasure of information lowers the entropy of the overall system and thus is accompanied by heat dissipation to the surrounding. Bennett further utilized Landauer’s argument to explain Maxwell’s demon to avoid a paradox violating the second law of thermodynamics [3,4]. Following the original work of Landauer, there are numerous analytical [5,6] and experimental studies [7,8,9,10,11,12,13] focused on the minimal energy consumption related to information processing.

A recent research interest is on analyzing erasure mechanisms that result in heat dissipation lower than $k_{B}T\ln2$ (which is the Landauer’s bound). In this regard, researchers have studied the effect of uncertainty in the erasure process and have shown that partially successful erasures result in heat dissipation lower than the Landauer’s bound [14,15]. It is shown in [14,15] that the lower bound on the heat dissipation is given by $k_{B}T\left(\ln2+p\ln p+\left(1-p\right)\ln\left(1-p\right)\right)$, termed the Generalized Landauer Bound (GLB), where *p* is the probability of reliability of the erasure process. An interesting observation reached by analyzing GLB is that a slight compromise on accuracy (by about 10%) of the erasure process holds the potential for lowering the associated minimum heat dissipation significantly (by about 50%). An important assumption made in arriving at the GLB in [5,12,13] is that there is ”insignificant” overlap between the two physical states that realize the single bit memory, that is, the two states of a memory bit have sufficient “physical separation”. Such an assumption is implicit in the experimental studies as well [7,8,9,10,11]. The effect of reduction in physical dimension on energetic and reliability aspects is not well established. In this article, we analyze the effect of overlap of the two wells in a single bit memory with regards to heat dissipation in erasure of information as well as reliability of stored information. We study the relationship between the Generalized Landauer Bound (GLB) and the average time from either well to cross the barrier at the origin with regards to the physical separation between the two states of a one bit memory (that is, “size” of the memory bit). Our approach introduces an “overlap parameter” and uses properties of mixture of Gaussian distributions to derive bounds on change in entropy associated with information erasure as well as average time of loss of stored information. The derived bounds on the entropy change converge to the GLB when the overlap between the two states become “insignificant” and hence is consistent with existing results in the literature [5,14,15]. We quantify the relationship between the overlap and the reduction in entropy change and utilize it to arrive at GLB. Here, we derive sub-Gaussian upper bounds analytically as opposed to the numerical study of entropy approximations for bit erasure in [16]. Furthermore, we obtain complimentary lower bounds on the decrease in thermodynamic entropy, demonstrating that these bounds are reasonably sharp, and, for bi-stable wells, physically separated by lengths close to their standard deviation, the error in entropy approximation incurred by the “insignificant overlap” approximation is significant. These quantitative results are of immediate application as they allow a tight approximation of the change in entropy in erasure process, relevant to the precise estimation of the associated minimal heat dissipation. A quantitative analysis is also provided for the case when the two states of memory are non identical and the erasure process moves the state into the well with higher/lower volume. Furthermore, we use mean first passage time [17] results to quantify the relationship between reliability of stored information in a memory bit and the overlap parameter. We quantify that a trade-off exists between lowering the minimum heat dissipation in information erasure and improving reliability of information stored in a single bit memory. Moreover, we determine a threshold on the overlap parameter, where a value higher than the threshold has minor benefits from the energetics as well as reliability standpoint. The threshold value can be used to determine memory densities that strike a good trade-off between reliability and thermodynamic cost of computations.

Our article provides analytical results that are very pertinent and timely to the current research focus of studying fundamentals of energetics of computations. The article aims to understand the effects of reducing size of fundamental building blocks of information storage devices on the thermodynamic and reliability aspects of computations. In our analysis, a smaller value of the overlap parameter implies a smaller size of the memory bit. The change in entropy bounds derived would also be applicable to enable analysis of other processes in the field of thermodynamics of information involving a mixture of Gaussian distributions—for example, in feedback processes where mutual information between system state and measurement is a key quantity of interest [18]. Moreover, the derived bounds on change in entropy have shown to be applicable in information theory [19], for analyzing capacity bounds of a channel transmitting discrete values corrupted by independent Gaussian noise. The analysis is also motivated by new paradigms of computation—for example, in stochastic computation [20] and neuromorphic memory architectures [21], where uncertainty of the success of computation is allowed and modeled a priori.

## 2. Single Bit Memory and Erasure Process with Uncertainty

### 2.1. Single Bit Memory

Following the original work of [1], we consider a Brownian particle in a double well potential as a model for a single bit memory. The location of the particle, *x*, in either well designates the state of the memory as 0 or 1. In this article, if the particle is located in the left well (x<0), we denote the state of memory as 0 and if the particle is located in the right well (x≥0), we denote the state as 1. The particle in either well is in thermal equilibrium with the surrounding whose temperature is assumed to be a constant *T*. For most of this article, we assume that both wells are identical unless specified.

In recent experimental validations of Landauer’s principle [7,8,11,22], it is seen that the double well potential in the neighborhood of the two stable states can be approximated as a convex quadratic function locally. It then follows from the canonical distribution expression that the equilibrium probability distribution is approximately a Gaussian distribution around the minimum of each of the well. Henceforth, we assume that the equilibrium probability distributions of the particle in the left and right wells are $N(-\mu ,\sigma^{2})$ and $N(\mu ,\sigma^{2}),$ respectively, where $N(\mu ,\sigma^{2})$ denotes a Normal distribution with mean μ and variance $\sigma^{2}$. Thus, if the memory is in state 0, the equilibrium probability distribution of the particle is $f_{0}\left(x\right)=Ce^{-{\left(x+\mu \right)}^{2}/2\sigma^{2}}$ and if the memory state is 1, the equilibrium probability distribution of the particle state is $f_{1}\left(x\right)=Ce^{-{\left(x-\mu \right)}^{2}/2\sigma^{2}}$, where *x* denotes the position of the particle and *C* is the normalization constant. This assumption is consistent with the notion of memory as described in [23], where a single bit memory is defined as a system with two stable states, such that the system is locally in equilibrium in either state and stays in one of the stable states (information retention) for the duration the memory bit is valid (related to the exit time across a barrier).

In this article, we study the effect of overlap between the two wells of the double well potential (that is, the effect of overlap between the two equilibrium probability distributions of the two states of a single bit memory) on the Landauer’s bound. The two distributions intersect at x=0, where $f_{0}\left(0\right)=f_{1}\left(0\right)=Ce^{-\alpha^{2}/2}$ with α:=μ/σ. Thus, the overlap between the two distributions is characterized by the parameter α, which is referred to as the overlap parameter. The higher (lower) the value of α, the lower (greater) is the overlap between the two equilibrium distributions. Moreover, α is also indicative of the aspect ratio of the memory bit as well as the physical extent of the memory bit for a known standard deviation of the equilibrium probability distributions.

Prior to erasure, it is equally likely for the state of the memory, *M*, to be zero or one, that is, $P\left(M=0\right)=P\left(M=1\right)=\frac{1}{2}$. The probability of finding the Brownian particle between *x* and x+dx is given by

(1) $$\begin{array}{cc} P(X\in (x,x+dx)) & =P(M=0)P(X\in (x,x+dx)|M=0)\;\\ & +P(M=1)P(X\in (x,x+dx)|M=1)\;\\ & =\frac{1}{2}f_{0}\left(x\right)dx+\frac{1}{2}f_{1}\left(x\right)dx. \end{array}$$

**Figure 2:**
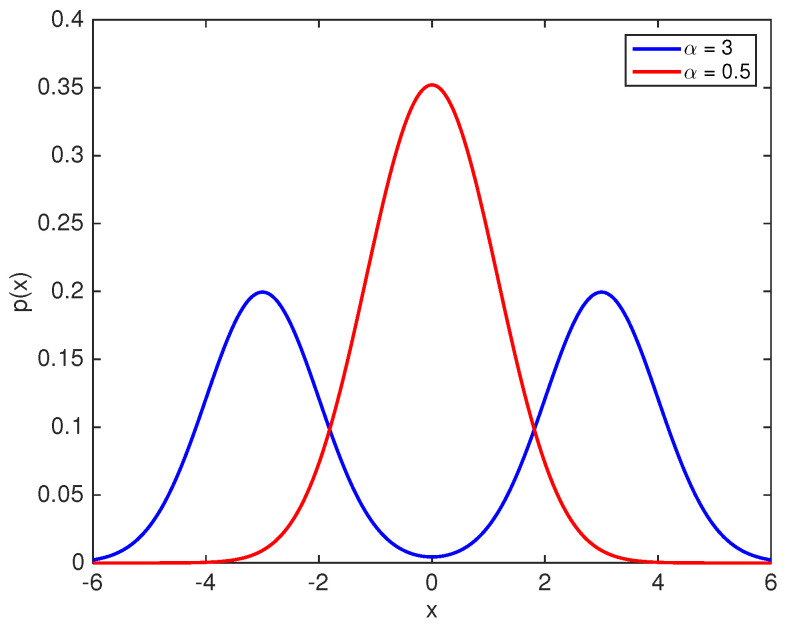
Probability distribution for α=0.5 and α=3.

**Figure 3:**
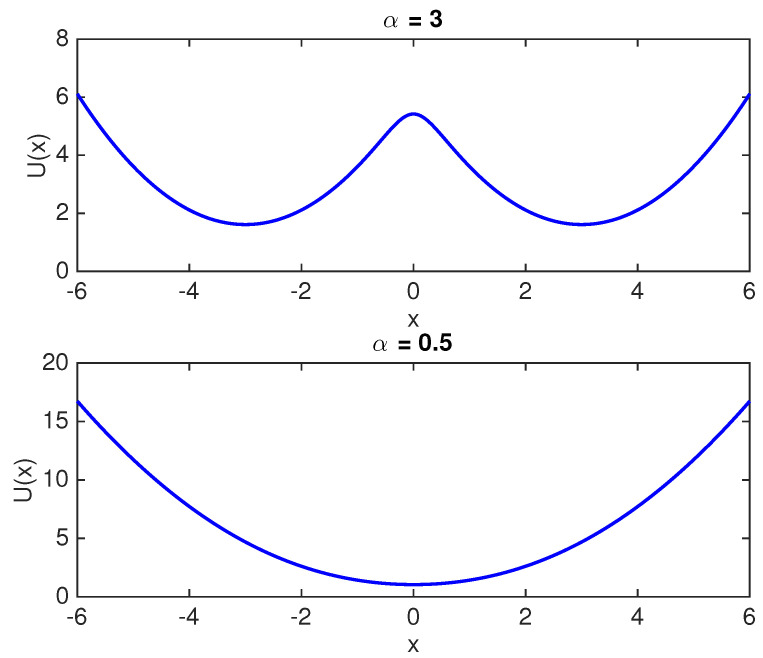
Potential U(x) landscape for α=0.5 and α=3. The potential U(x) is computed using the Canonical distribution relation, $p\left(x\right)=\frac{e^{-U\left(x\right)/k_{B}T}}{Z}$, where Z denotes the normalization constant.

Thus, the probability distribution function, f(x), of the particle prior to undergoing an erasure process is an equally weighted mixture of $f_{0}\left(x\right)$ and $f_{1}\left(x\right)$. In order to represent a valid single bit memory, this distribution must be a bi-modal distribution (due to a symmetric double well stable potential). In this regard, an interesting result is that: an equally weighted mixture of symmetric Gaussian distributions is uni-modal if and only if α≤1; otherwise, it is bi-modal [24]. Thus, in order to represent a symmetric single bit memory with two well defined states, f(x) needs to be a bi-modal distribution with the two modes being alike. Hence, a single bit memory must have α>1 in order to be a valid memory bit. Figure 2 and Figure 3 show the probability distribution f(x) and potential U(x) respectively for α=3 and α=0.5. It is seen that the distribution is uni-modal (the corresponding potential is a single well potential) for α=0.5 and cannot be used to realize the two states of a single bit memory. However, α=3 results in a symmetric bi-modal distribution and a symmetric double well potential as seen in Figure 3, which results in a well defined memory bit. It is important to note that the overlap parameter α depends on the physical design of the memory bit (the double well potential precisely). Recent studies on Landauer’s bound [7,8,11,22] used α≈20.

Based on the above model of a single bit memory, there is a non zero probability to commit an error in judging the state of the memory, *M*, from the measured position of the Brownian particle *x*. This is because P(M=0|X∈(x,x+dx))>0 for all x∈R, which implies that there is a non zero probability for the memory *M* to be in state 0 or 1, irrespective of any value of the position of the Brownian particle. Consider a threshold T such that, if x<T, then we infer M=0; otherwise, M=1. The mis-classification error probability, P(E), is given as

(2) $$\begin{array}{c} P\left(E\right)=\frac{1}{2}\int_{x<T}e^{-{\left(x-\mu \right)}^{2}/2\sigma^{2}}dx+\frac{1}{2}\int_{x>T}e^{-{\left(x+\mu \right)}^{2}/2\sigma^{2}}dx. \end{array}$$

It is shown in [25] that P(E) is minimum if T=0. In the rest of the manuscript, we choose T=0, which is an optimal choice and is also used in recent experimental studies on the Laundauer’s principle [7,8,11]. P(E) is a quantifier of the loss of information which approaches zero if the overlap parameter α:=μ/σ approaches *∞*. This means that, as the overlap between the two wells becomes insignificant, the loss of information vanishes and the state of the memory bit can be identified accurately from a position of the Brownian particle. We now analyze the effect of the overlap parameter on energetics of erasure of one bit of information.

### 2.2. Erasure with Uncertainty

**Figure 4:**
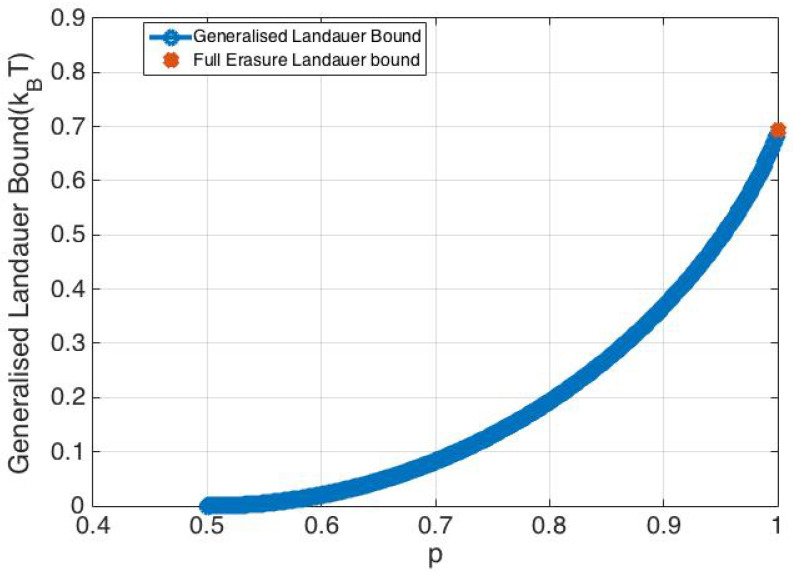
Generalized Landauer Bound (GLB) as a function of protocol reliability parameter *p*.

Erasure is a process where, irrespective of the initial state of the memory bit, the final state is zero (also known as *reset to zero*). In a double well, potential representation of a single bit memory erasure entails that the particle needs to be transferred to the left well, irrespective of the initial position of the particle. Erasure is achieved using a particle transfer protocol (also referred as erasure protocol), which reliably moves the Brownian particle to the left of the origin. We associate reliability parameter, *p*, with the erasure protocol, where, p:=P(M=0) after application of the erasure protocol. The Generalized Landauer Bound (GLB) from [7,14,15] states that, if the reliability parameter of the erasure process is *p*, then the associated average heat dissipation is at least $k_{B}T\left(\ln2+p\ln p+\left(1-p\right)\ln\left(1-p\right)\right)$. The GLB evaluates to $k_{B}T\ln2$ for p=1; to 0 for p=0.5 and is shown in Figure 4 as a function of the reliability parameter *p*. Note that p<0.5 is not considered here as it would imply a reset to one operation. It is important to point out that the reliability parameter, *p*, depends on the specific protocol used for accomplishing the erasure and will be referred to as the protocol reliability parameter. We would like to bring the attention of the reader to [7,11,26], where the authors discuss the dependence of protocol reliability parameter on the speed of the erasure process.

Under the assumption of α>1, we analyze the effect of overlap parameter α on the Generalized Landauer Bound. The probability distribution function of the particle before undergoing erasure, f(x), is given by, $f\left(x\right)=\frac{1}{2}f_{0}\left(x\right)+\frac{1}{2}f_{1}\left(x\right)$ (see Equation (1)). After applying an erasure protocol to the memory bit with reliability parameter *p*, the probability of finding a particle between *x* and x+dx is given by,

(3) $$\begin{array}{c} P\left(X\in \left(x,x+dx\right)\right)=pf_{0}\left(x\right)dx+\left(1-p\right)f_{1}\left(x\right)dx. \end{array}$$

**Figure 5:**
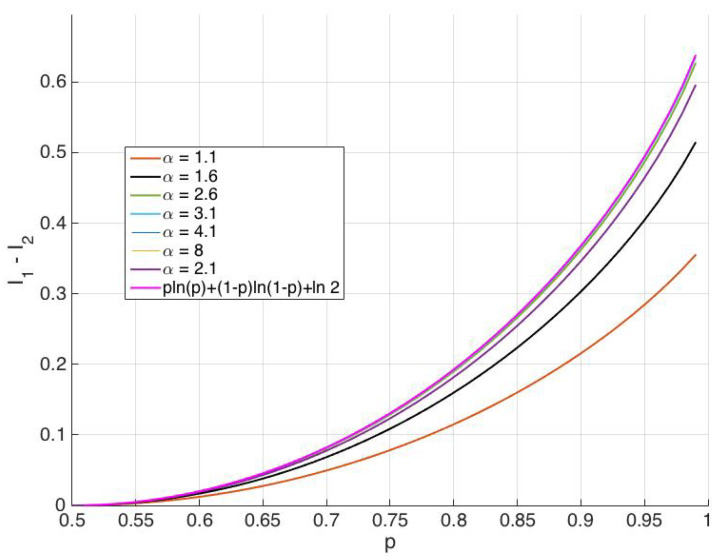
$I_{1}-I_{2}$ as a function of *p* for various values of α. $I_{1}-I_{2}$ approaches plnp+(1−p)ln(1−p)+ln2 (pink curve) as α increases.

Let $g\left(x\right):=pf_{0}\left(x\right)+\left(1-p\right)f_{1}\left(x\right)$. The thermodynamic entropy of the system before erasure is $S_{f}=-k_{B}\int_{-\infty }^{\infty }f\left(x\right)\ln\left(f\left(x\right)\right)dx$, and, after undergoing erasure process using a protocol with reliability parameter *p*, is $S_{g}=-k_{B}\int_{-\infty }^{\infty }g\left(x\right)\ln\left(g\left(x\right)\right)dx$. It follows from the 2nd Law of Thermodynamics that the average heat dissipation,

$$\begin{array}{c} \langle Q_{d}\rangle \geq T\left(S_{f}-S_{g}\right)=k_{B}T\left(I_{1}-I_{2}\right), \end{array}$$

where $I_{1}=\alpha^{2}-\frac{1}{\sqrt{2\pi }\alpha }e^{-\frac{\alpha^{2}}{2}}\int_{-\infty }^{\infty }e^{-\frac{x^{2}}{2\alpha^{2}}}\cosh\left(x\right)\ln\left(\cosh\left(x\right)\right)dx$ and $I_{2}=\alpha^{2}-\frac{1}{\sqrt{2\pi }\alpha }e^{-\frac{\alpha^{2}}{2}}\int_{-\infty }^{\infty }e^{-\frac{x^{2}}{2\alpha^{2}}}\left(pe^{-x}+\left(1-p\right)e^{x}\right)\ln\left(pe^{-x}+\left(1-p\right)e^{x}\right)dx$. The dependence of $I_{1}-I_{2}$ on the overlap parameter α is shown in Figure 5. It is seen that $k_{B}T\left(I_{1}-I_{2}\right)$ approaches the GLB for large values of the overlap parameter α. Note that, for α≤2.1, the decrease in thermodynamic entropy $k_{B}\left(I_{1}-I_{2}\right)$ is less than $k_{B}\left(p\ln p+\left(1-p\right)\ln\left(1-p\right)+\ln2\right)$ and is well approximated by $k_{B}\left(p\ln p+\left(1-p\right)\ln\left(1-p\right)+\ln2\right)$, when α>5. As seen from Figure 5, for a given protocol reliability parameter *p* and 1<α<5, the associated minimum average heat dissipation due to erasure is lower than the GLB. Hence, allowing overlap (by reducing the physical separation) between the two states of the memory bit can enable energy dissipation lower than the GLB in an erasure process.

We remark that the protocol reliability parameter *p* is dependent on the protocol used for performing erasure and the overlap parameter α of the memory bit. There is a subtle difference between the protocol reliability parameter *p* and the probability of success of an erasure, $p_{s}$, and is discussed below.

**Figure 6:**
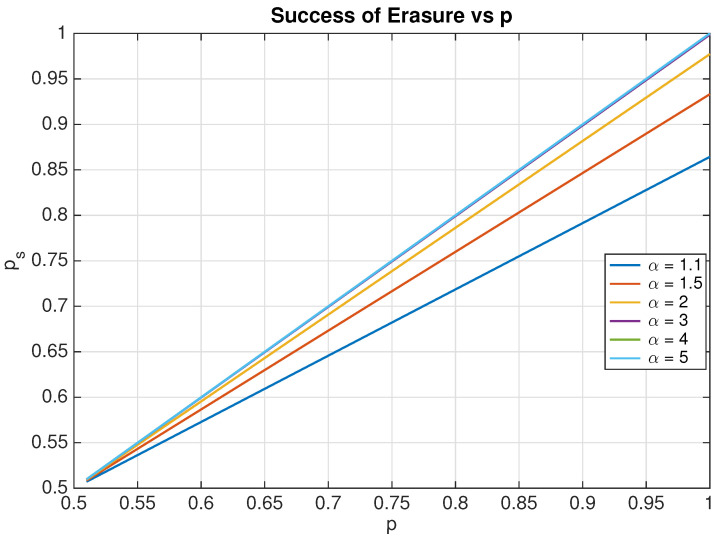
Variation of probability of success of erasure as a function of the overlap parameter and protocol reliability parameter *p*.

Without loss of generality, it can be assumed that, if the position of the particle is to the left of the origin, the state of the memory is deemed as 0; the erasure process is considered successful if, after application of the erasure protocol the position of the particle, *x*, is observed to be less than zero. Thus, the probability of success, $p_{s}$, of the erasure process is given by

(4) $$\begin{array}{cc} p_{s}=P\left(X<0\right) & =p\int_{-\infty }^{0}\frac{1}{\sqrt{2\pi \sigma^{2}}}e^{-\frac{{\left(x+\mu \right)}^{2}}{2\sigma^{2}}}dx+\left(1-p\right)\int_{-\infty }^{0}\frac{1}{\sqrt{2\pi \sigma^{2}}}e^{-\frac{{\left(x-\mu \right)}^{2}}{2\sigma^{2}}}dx\;\\ & =p\int_{-\infty }^{\alpha }\frac{1}{\sqrt{2\pi }}e^{-\frac{u^{2}}{2}}du+\left(1-p\right)\int_{-\infty }^{-\alpha }\frac{1}{\sqrt{2\pi }}e^{-\frac{v^{2}}{2}}dv,u:=\frac{x}{\sigma }+\alpha ,v:=\frac{x}{\sigma }-\alpha ,\;\\ & =\frac{1}{2}\mathrm{erfc}\left(\alpha /\sqrt{2}\right)+p\times \mathrm{erf}\left(\alpha /\sqrt{2}\right), \end{array}$$

where erf and erfc denote the Gaussian error and complementary error function. Thus, in the limit $\alpha \to \infty ,p_{s}\to p$. In Figure 6, we plot $p_{s}$ by varying α and *p*. It is seen that, for α≥3, $p_{s}$ is very close to *p*. Thus, $p_{s}$ can be considered as a good approximation of *p* for α≥3. Empirically, $p_{s}$ is determined by applying the specified protocol to several realizations of the memory bit and then observing what fraction of those resulted in the particle being located to the left of the origin after the completion of the protocol [7,8,11]. Once an estimate of $p_{s}$ is known, with the knowledge of α, the protocol reliability parameter can be estimated.

In the next section, we derive upper and lower bounds to the change in entropy as a function of the protocol reliability parameter *p* and overlap parameter α.

## 3. Effect of Overlap Parameter on Thermodynamic Cost of Erasure

### 3.1. Upper and Lower Bounds on the Change in Entropy during Erasure

We will establish in this section that the following upper and and lower bounds on the decrease in entropy, $S_{f}-S_{g}$,

(5) $$\begin{array}{cc} S_{f}-S_{g} & \leq k_{B}\left(\ln2-H\left(p\right)-\ln\left(1+e^{-2\alpha^{2}}\right)+C\left(2-H\left(p\right)+4\alpha^{2}\right)e^{-\alpha^{2}/2}\right),\;\mathrm{and}, \end{array}$$

(6) $$\begin{array}{cc} S_{f}-S_{g} & \geq k_{B}(\ln2-H\left(p\right)-C\left(2+4\alpha^{2}+\ln2\right)e^{-\alpha^{2}/2}+p\ln\left(1+\frac{1-p}{p}e^{-2\alpha^{2}}\right)+\;\\ & \left(1-p\right)\ln\left(1+\frac{p}{1-p}e^{-2\alpha^{2}}\right)). \end{array}$$

Here, H(p):=−plnp−(1−p)ln(1−p). Consider a probability distribution function (pdf), $g\left(.\right):=pN\left(-\mu ,\sigma^{2}\right)+\left(1-p\right)N\left(\mu ,\sigma^{2}\right)$, and another pdf, $\bar{g}\left(.\right):=pN\left(-\alpha ,1\right)+\left(1-p\right)N\left(\alpha ,1\right)$, where α is the overlap parameter $\frac{\mu }{\sigma }$. Then, $S_{g}=S_{\bar{g}}+k_{B}\ln\sigma$ [27]. Similarly, for $p=\frac{1}{2}$, with $f\left(.\right)=\frac{1}{2}N\left(-\mu ,\sigma^{2}\right)+\frac{1}{2}N\left(\mu ,\sigma^{2}\right)$ and $\bar{f}\left(.\right):=\frac{1}{2}N\left(-\alpha ,1\right)+\frac{1}{2}N\left(\alpha ,1\right),$ we have $S_{f}=S_{\bar{f}}+k_{B}\ln\sigma .$ Thus, $S_{f}-S_{g}=S_{\bar{f}}-S_{\bar{g}}$. In the derivations below, we are interested in the change in entropy between an initial pdf described by *f* and a final distribution described by *g*. It is evident from the relation above that we can limit our discussion to g=pN(−α,1)+(1−p)N(α,1) and $f=\frac{1}{2}N\left(-\alpha ,1\right)+\frac{1}{2}N\left(\alpha ,1\right).$ We also use $f_{0}\left(x\right)=Ce^{-\frac{\left(x+\alpha^{2}\right)}{2}}$ and $f_{1}\left(x\right)=Ce^{-\frac{\left(x-\alpha^{2}\right)}{2}}$, where $C=\frac{1}{\sqrt{2\pi }}$.

For the derivation below, it is assumed that p∈[0.5,1) and α>1.

It follows (see Appendix A) that

(7) $$\begin{array}{c} \int_{-\infty }^{\infty }pf_{0}\left(x\right)\ln\left(pf_{0}\left(x\right)+\left(1-p\right)f_{1}\left(x\right)\right)dx-\;\\ \int_{-\infty }^{\infty }pf_{0}\left(x\right)\ln\left(pf_{0}\left(x\right)\right)dx\;\\ <C\left(2\left(1-p\right)+p\ln\left(\frac{e^{4\alpha^{2}}}{p}\right)\right)e^{-\alpha^{2}/2}. \end{array}$$

Similarly, one can show that

(8) $$\begin{array}{c} \int_{-\infty }^{\infty }\left(1-p\right)f_{1}\left(x\right)\ln\left(pf_{0}\left(x\right)+\left(1-p\right)f_{1}\left(x\right)\right)dx\;\\ -\int_{-\infty }^{\infty }\left(1-p\right)f_{1}\left(x\right)\ln\left(\left(1-p\right)f_{1}\left(x\right)\right)dx\;\\ <C\left(2p+\left(1-p\right)\ln\left(\frac{e^{4\alpha^{2}}}{1-p}\right)\right)e^{-\alpha^{2}/2}. \end{array}$$

Let $K:=\int_{-\infty }^{\infty }f_{0}\left(x\right)\ln\left(f_{0}\left(x\right)\right)dx=\int_{-\infty }^{\infty }f_{1}\left(x\right)\ln\left(f_{1}\left(x\right)\right)dx.$

Using Equations (7) and (8) with $p=\frac{1}{2}$ leads to the following lower bound on $S_{f}$,

(9) $$\begin{array}{cc} S_{f} & \geq k_{B}\left(-K+\ln2-C\left(2+4\alpha^{2}+\ln2\right)e^{-\alpha^{2}/2}\right). \end{array}$$

From Equations (7) and (8), it also follows that

(10) $$\begin{array}{cc} -S_{g} & \leq k_{B}(p\ln p+\left(1-p\right)\ln\left(1-p\right)+K\;\\ & +C\left(2+p\ln p+\left(1-p\right)\ln\left(1-p\right)+4\alpha^{2}\right)e^{-\alpha^{2}/2}). \end{array}$$

In the Appendix A, we also derive the following lower bounds,

(11) $$\begin{array}{c} \int_{-\infty }^{\infty }pf_{0}\left(x\right)\ln\left(pf_{0}\left(x\right)+\left(1-p\right)f_{1}\left(x\right)\right)dx-\int_{-\infty }^{\infty }pf_{0}\left(x\right)\ln\left(pf_{0}\left(x\right)\right)dx\geq p\ln\left(1+\frac{1-p}{p}e^{-2\alpha^{2}}\right),\mathrm{and}, \end{array}$$

(12) $$\begin{array}{c} \int_{-\infty }^{\infty }\left(1-p\right)f_{1}\left(x\right)\ln\left(pf_{0}\left(x\right)+\left(1-p\right)f_{1}\left(x\right)\right)dx-\int_{-\infty }^{\infty }\left(1-p\right)f_{1}\left(x\right)\ln\left(\left(1-p\right)f_{1}\left(x\right)\right)dx\;\\ \geq \left(1-p\right)\ln\left(1+\frac{p}{1-p}e^{-2\alpha^{2}}\right). \end{array}$$

Using Equations (11) and (12) with p=1/2, we obtain the following upper bound on $S_{f}$,

(13) $$\begin{array}{cc} S_{f} & \leq k_{B}\left(-K+\ln2-\ln\left(1+e^{-2\alpha^{2}}\right)\right). \end{array}$$

Furthermore, from Equations (11) and (12), we obtain the following lower bound on $-S_{g}$,

(14) $$\begin{array}{c} -S_{g}\geq k_{B}\left(K+p\ln p+\left(1-p\right)\ln\left(1-p\right)+p\ln\left(1+\frac{1-p}{p}e^{-2\alpha^{2}}\right)+\left(1-p\right)\ln\left(1+\frac{p}{1-p}e^{-2\alpha^{2}}\right)\right). \end{array}$$

It follows from Equations (9) and (13) that

(15) $$\begin{array}{c} k_{B}\left(-K+\ln2-C\left(2+4\alpha^{2}+\ln2\right)e^{-\alpha^{2}/2}\right)\;\\ \leq S_{f}\leq k_{B}\left(-K+\ln2-\ln\left(1+e^{-2\alpha^{2}}\right)\right). \end{array}$$

Similarly, it follows from Equations (10) and (14) that

(16) $$\begin{array}{c} k_{B}\left(K-H\left(p\right)+p\ln\left(1+\frac{1-p}{p}e^{-2\alpha^{2}}\right)+\left(1-p\right)\ln\left(1+\frac{p}{1-p}e^{-2\alpha^{2}}\right)\right)\;\\ \leq -S_{g}\leq k_{B}\left(K-H\left(p\right)+C\left(2-H\left(p\right)+4\alpha^{2}\right)e^{-\alpha^{2}/2}\right), \end{array}$$

where H(p)=−plnp−(1−p)ln(1−p). Using Equations (15) and (16), it is concluded that the difference between the initial and final entropy, $S_{f}-S_{g}$, satisfies the bounds in Equations (5) and (6).

The case of p=1 has to be considered separately. If p=1, $g\left(x\right)=f_{0}\left(x\right)$ and $-S_{g}=k_{B}K$. It then follows from Equations (9) and (13) that

$$\begin{array}{cc} S_{f}-S_{g} & \geq k_{B}\left(\ln2-C\left(2+4\alpha^{2}+\ln2\right)e^{-\alpha^{2}/2}\right),\\ S_{f}-S_{g} & \leq k_{B}\left(\ln2-\ln\left(1+e^{-2\alpha^{2}}\right)\right). \end{array}$$

In the case p=1 as well, it follows that $\lim_{\alpha \to \infty }S_{f}-S_{g}=k_{B}\ln\left(2\right)$, which is the Landauer’s bound.

### 3.2. Relationship to the Generalized Landauer Bound

**Figure 7:**
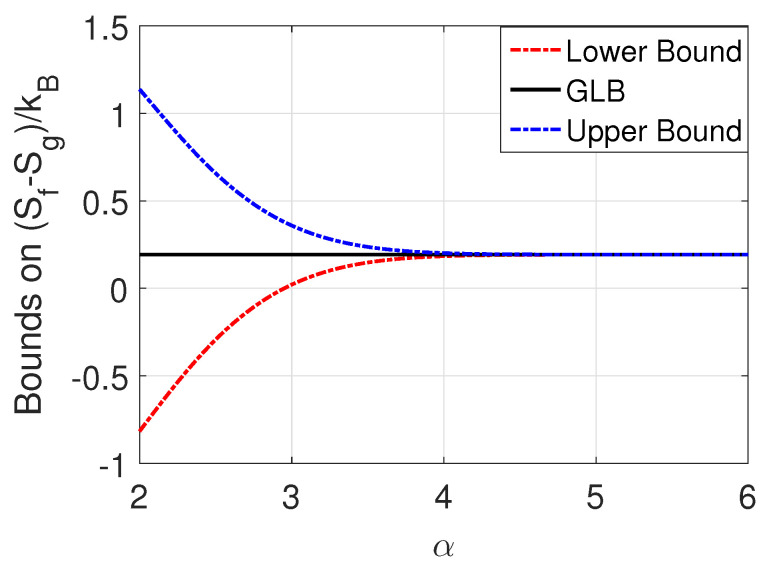
Lower and Upper bounds of $\left(S_{f}-S_{g}\right)/k_{B}$ and the GLB as a function of α for p=0.8.

Note from Equations (5) and (6), $\lim_{\alpha \to \infty }S_{f}-S_{g}=k_{B}\left[\ln\left(2\right)+p\ln\left(p\right)+\left(1-p\right)\ln\left(1-p\right)\right]$(GLB), where the upper and lower bounds on the change in entropy, both converge to the GLB. The convergence to the limit is with respect to the overlap parameter α is exponentially fast for both the upper and lower bound. Thus, in the limiting case of α→∞, it follows from the 2nd Law of Thermodynamics that $\frac{\langle Q_{d}\rangle }{T}\geq k_{B}\left[\ln\left(2\right)+p\ln\left(p\right)+\left(1-p\right)\ln\left(1-p\right)\right]$, implying that for a quasi static erasure using a protocol with reliability parameter *p*, $\langle Q_{d}\rangle =k_{B}T\left[\ln\left(2\right)+p\ln\left(p\right)+\left(1-p\right)\ln\left(1-p\right)\right]$. In Figure 7, we present the derived lower and upper bounds on $\left(S_{f}-S_{g}\right)/k_{B}$ as a function of the overlap parameter for p=0.8. The exponential convergence to the GLB value for p=0.8 is evident. Table 1 lists the difference between the upper (Equation (5)) and lower (Equation (6)) bounds of $\left(S_{f}-S_{g}\right)/k_{B}$ for various values of the overlap parameter, α, and protocol reliability parameter, *p*. It is seen that the difference is significant for α<5 and is almost zero for α=5 (insignificant overlap). Here, it is seen that, with a threshold of 5 for the overlap parameter α, increasing α beyond the threshold accrues only marginal gains energetically. The recent experimental studies on verification of the Landauer’s bound [7,8,11] employed α≈20, indicating that there is considerable scope to increase the density of the resulting memory.

### 3.3. Extensions to Asymmetric 1 Bit Memory

An asymmetric one bit memory and the associated minimum heat dissipation for its erasure is discussed in [10,23]. In particular, Ref. [10] presents an experimental study of the minimum heat dissipation for perfect erasures of a bit of asymmetric memory, with one well being wider than the other, and considers the two cases of resetting the bit into the wider and narrower well. Motivated from the discussion in [10], we extend the GLB to the case of non-identical wells, where one well is wider than the other.

We assume that, initially, the particle has equal probability to be in either well and the initial probability distribution of the particle is given by f(x) as described earlier (see Equation (1)). Similarly, for erasures using a protocol with reliability parameter *p*, the final probability distribution of the particle is given as g(x) (see Equation (3)).

#### 3.3.1. Erasing into Low Entropy Well

Consider $f_{0}\left(x\right)=Ce^{-\frac{{\left(x+\mu \right)}^{2}}{2\sigma^{2}}}$ and $f_{1}\left(x\right)=\frac{C}{\beta }e^{-\frac{{\left(x-\mu \right)}^{2}}{2{\left(\beta \sigma \right)}^{2}}}$ with β>1. The particle has higher entropy in state 1 as compared to state 0. In this case, for ‘reset to zero’ with protocol reliability parameter *p*, one can show that

(17) $$\begin{array}{c} k_{B}\left(-\frac{C}{2}\left(2+\frac{9\alpha^{2}}{2}+\ln2\right)e^{-\alpha^{2}/2}-\frac{C}{2}\left(2+\ln2+\ln\beta +4\alpha^{2}\right)e^{-\alpha^{2}/2\beta^{2}}\right)\;\\ \leq S_{f}-S_{g}-k_{B}\left(\ln2+p\ln p+\left(1-p\right)\ln\left(1-p\right)+\left(\frac{1}{2}-\left(1-p\right)\right)\ln\beta \right)\leq \;\\ k_{B}\left(C\left(2p+p\ln\left(\frac{e^{9\alpha^{2}/2}}{p}\right)\right)e^{-\alpha^{2}/2}+C\left(2\left(1-p\right)+\left(1-p\right)\ln\left(\frac{\beta e^{4\alpha^{2}}}{1-p}\right)\right)\right). \end{array}$$

**Figure 8:**
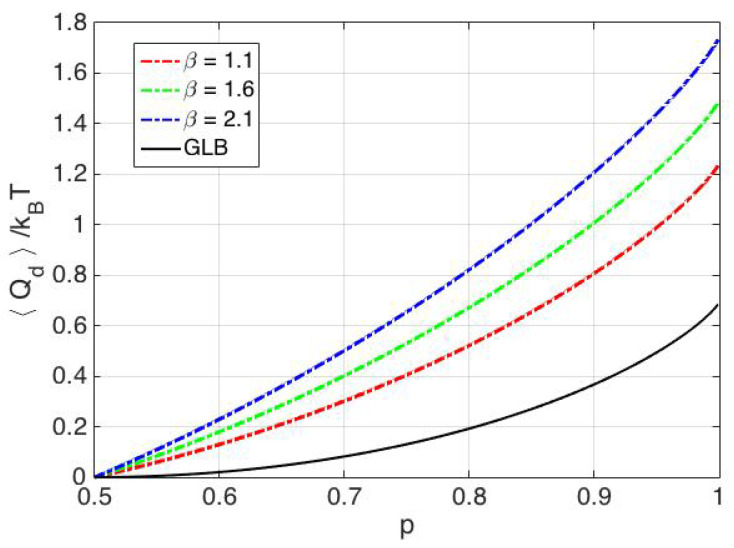
Minimum heat dissipation associated with erasing into a low entropy well in a quasi static manner.

Thus, $\lim_{\alpha \to \infty }S_{f}-S_{g}=k_{B}\left[p\ln p+\left(1-p\right)\ln\left(1-p\right)+\left(\frac{1}{2}-\left(1-p\right)\right)\ln\beta +\ln2\right]$. The rate of convergence is exponential with respect to the parameter α and depends inversely on the parameter β. Thus, in the limiting case of the two wells being sufficiently far apart and a quasi-static erasure process with protocol reliability parameter *p*, $\langle Q_{d}\rangle =k_{B}T\left[p\ln p+\left(1-p\right)\ln\left(1-p\right)+\left(\frac{1}{2}-\left(1-p\right)\right)\ln\beta +\ln2\right]$. Here, the bit was erased into the well with lower entropy, hence the limiting value of the change in entropy is higher than the identical wells case. In Figure 8, the heat dissipation associated with erasing into the low entropy well is shown for various values of the asymmetry parameter β and is compared with the Generalized Landauer Bound. It is seen that, as the asymmetry parameter is increased, the associated heat dissipation also increases for various protocol reliability parameters. The case of erasing into the high entropy well is more relevant and is presented next.

#### 3.3.2. Erasing into a High Entropy Well

**Figure 9:**
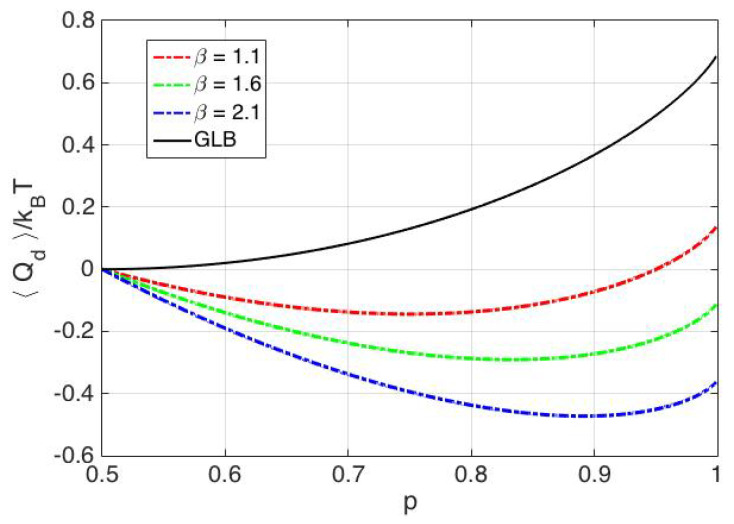
Minimum heat dissipation associated with erasing into high entropy well in a quasi static manner.

Consider erasing into the higher entropy well, that is, $f_{1}\left(x\right)=Ce^{-\frac{{\left(x-\mu \right)}^{2}}{2\sigma^{2}}}$ and $f_{0}\left(x\right)=\frac{C}{\beta }e^{-\frac{{\left(x+\mu \right)}^{2}}{2{\left(\beta \sigma \right)}^{2}}}$ with β>1. Here, the particle has higher entropy in the state 0 as compared to state 1. In this case, for ‘reset to zero’ with protocol reliability parameter *p*, one can arrive at inequalities like the previous case by replacing β with 1/β in Equation (17). It then follows that $\lim_{\alpha \to \infty }S_{f}-S_{g}=k_{B}\left[p\ln p+\left(1-p\right)\ln\left(1-p\right)+\left(\left(1-p\right)-\frac{1}{2}\right)\ln\beta +\ln2\right]$. In the limiting case of the two wells being sufficiently far apart, for a quasi-static process, $\langle Q_{d}\rangle =k_{B}T\left[p\ln p+\left(1-p\right)\ln\left(1-p\right)-\left(\frac{1}{2}-\left(1-p\right)\right)\ln\beta +\ln2\right]$. Here, the bit was erased into the well with higher entropy, hence the limiting value of the change in entropy is lower than the identical wells case. In Figure 9, the heat dissipation associated with erasing into the high entropy well is shown for various values of the asymmetry parameter β and is compared with the Generalized Landauer Bound. It is seen that, as the asymmetry parameter is increased, the associated heat dissipation decreases for various protocol reliability parameters. In this case, it is seen that erasure can be achieved without any associated heat dissipation. Hence, asymmetry can be utilized toward realizing logically irreversible computations with zero dissipation.

## 4. Effect of Overlap Parameter on the Reliability of a Memory Bit

In the previous sections, it is seen that a smaller value of the overlap parameter α results in lower minimum average heat dissipation for quasi static bit erasures. A smaller value of α implies a lower barrier height (U(0)−U(μ)), thereby resulting in transitions of the Brownian particle between the two wells. If such a transition occurs frequently, then the memory bit is considered unreliable. In this section, we quantify the degree of unreliability of a memory bit as a function of the overlap parameter α. Although the discussions in the previous sections are for quasi static erasures, the exit time results of this section are more relevant for erasures achieved in finite time [28].

We will use the mean exit time from the mean of a well (right or left) to the barrier (at the origin) as a metric to quantify the reliability of the bit. A desirable mean exit time from either well to the barrier is several years to ensure reliability of stored information in the memory bit. The mean exit time across the barrier (at x=0) from the mean of the right well (at x=μ), denoted by T(μ→0), (under the assumption of stochastic dynamics of the Brownian particle being governed by the overdamped Langevin equation [29]) is given as [30],

(18) $$\begin{array}{c} T\left(\mu \to 0\right)=\pi \sqrt{\frac{|U^{\prime \prime }\left(0\right)|}{U^{\prime \prime }\left(\mu \right)}}e^{\left(U\left(0\right)-U\left(\mu \right)\right)/k_{B}T}, \end{array}$$

where it follows from the canonical distribution that, $U\left(x\right)/k_{B}T=-\ln p(x)/Z$. Here, f(x) denotes the equilibrium probability density, which is given as $f\left(x\right)=\frac{1}{2}N\left(\mu ,\sigma \right)+\frac{1}{2}N\left(-\mu ,\sigma \right)$ and Z is the normalization constant. The barrier height, U(0)−U(α) is given as

(19) $$\begin{array}{c} \frac{U(0)-U(\mu )}{k_{B}T}=\ln\frac{f(\mu )}{f(0)}=\ln\frac{e^{\alpha^{2}/2}\left(1+e^{-2\alpha^{2}}\right)}{2}=\frac{\alpha^{2}}{2}+\ln\left(1+e^{-2\alpha^{2}}\right)-\ln2. \end{array}$$

**Figure 10:**
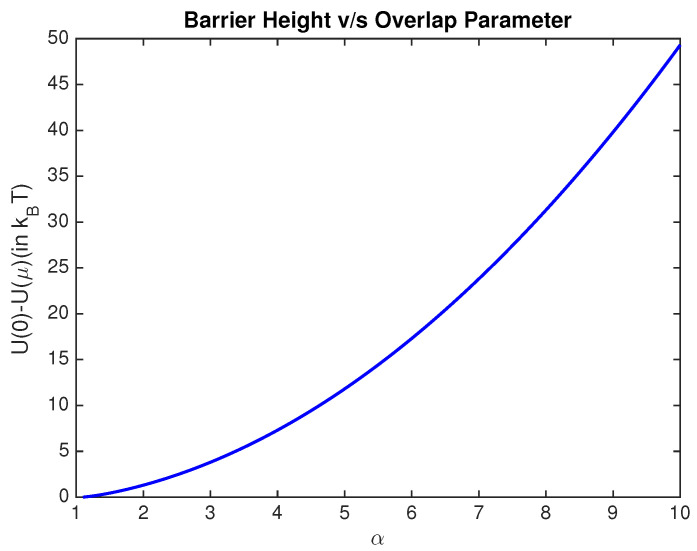
Barrier height in $k_{B}T$ as a function of the overlap parameter.

Thus, the barrier height, to a good approximation, is a quadratic function of the overlap parameter. In Figure 10, the increase in barrier height on increasing the overlap parameter is shown. It is evident that, as the overlap between the two wells decreases, the barrier height at the origin increases relative to the minima of the two wells and it is less likely to find the particle closer to the barrier.

Using Equation (19) and substituting the expressions for $U^{{}^{\prime \prime }}\left(\mu \right),U^{{}^{\prime \prime }}\left(0\right)$ in Equation (18) leads to,

(20) $$\begin{array}{c} T\left(\mu \to 0\right)=\frac{\pi }{2}\sqrt{\frac{\alpha^{2}-1}{1-\alpha^{2}{\mathrm{sech}}^{2}\left(\alpha^{2}/\sigma \right)}}e^{\alpha^{2}/2}\left(1+e^{-2\alpha^{2}}\right). \end{array}$$

**Figure 11:**
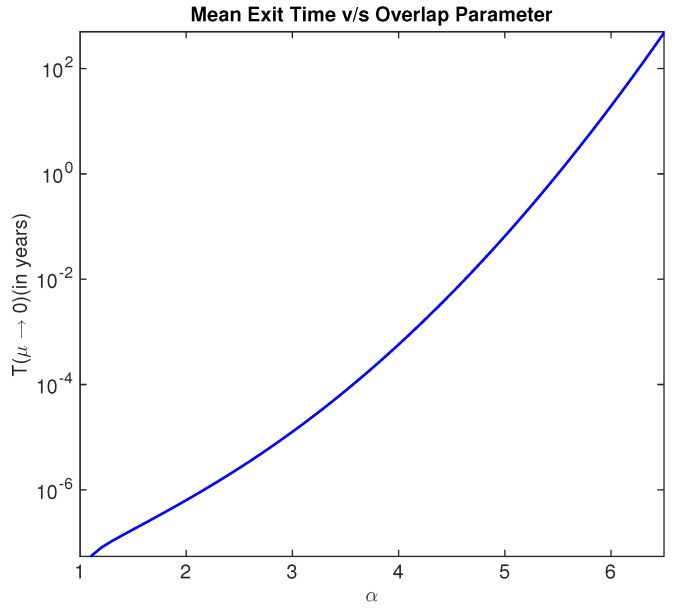
Mean exit time as a function of the overlap parameter for σ=1.

In Figure 11, we show the variation of mean exit time with respect to the overlap parameter α. It is seen that the mean exit time from the mean of the right well (or the left well) increases exponentially as α increases. It is seen that α≈7 has a mean exit of several hundred years. Based on our analysis, we conclude that reliability of the memory bit has exponential quadratic improvement while the minimum average heat dissipation has quadratic exponential decay to the GLB on increasing the overlap parameter α. Thus, the trade-off between energy dissipation in erasing information and reliability of the memory can be decided by a judicious choice of the overlap parameter.

## 5. Conclusions

We quantify the dependence of the decrease in entropy associated with erasure of a bit of information on the amount of overlap between the equilibrium distributions of the two states of a one bit memory. It is seen that overlap can lead to considerably lower heat dissipation as compared to the GLB in a quasi static erasure process. This is primarily due to loss of information, primarily in the region between the two wells. We quantified the effect of loss of information on the change in entropy associated with partial information erasure by deriving tight upper and lower bounds, which exponentially converge to the GLB when the physical separation between the two states is large (loss of information is 0). A conclusion is reached that α≈5 represents a threshold for energetics of computation associated with a single bit memory, where memory bit designs with α>5 have insignificant gains compared to α≈5 case from an energetics perspective. Furthermore, the effect of asymmetry between the two wells of a single bit memory on the associated minimum heat dissipation for erasure processes is analyzed. Finally, we used mean exit time across a barrier relationships to demonstrate the effect of the overlap parameter on the reliability of the bit. It is seen that a higher value of the overlap parameter results in a higher mean exit time and hence a more reliable memory. The slight change in the overlap parameter resulted in exponential improvement of the bit reliability. Thus, we arrive at a trade-off between minimizing heat dissipation and improving bit reliability by introducing overlap between the two wells of a memory bit. We showed that α≈7 resulted in bit reliability of several hundred years; beyond 7, the gains in reliability could be inconsequential. Thus, α≈7 can serve as a guide for designing memory with improved memory density, while retaining reliability as well as minimizing loss of information.

## Figures and Tables

**Table 1:** Difference between the upper (Equation (5)) and lower (Equation (6)) bounds of $\left(S_{f}-S_{g}\right)/k_{B}$ as a function of α and *p*.

	*p*	0.6	0.7	0.8
α				
1.5		2.829	2.838	2.852
2.0		1.944	1.947	1.953
2.5		0.946	0.948	0.949
3.0		0.336	0.337	0.338
5.0		3.033 × 10${}^{-4}$	3.034 × 10${}^{-4}$	3.035 × 10${}^{-4}$

## Appendix A

### Appendix A.1. Upper Bounds

First, we derive an upper bound for the difference,

$$\begin{array}{c} \int_{-\infty }^{\infty }pf_{0}\left(x\right)\ln\left(pf_{0}\left(x\right)+\left(1-p\right)f_{1}\left(x\right)\right)dx-\int_{-\infty }^{\infty }pf_{0}\left(x\right)\ln\left(pf_{0}\left(x\right)\right)dx, \end{array}$$

which is simplified as

(A1) $$\begin{array}{c} p\int_{-\infty }^{\infty }f_{0}\left(x\right)\ln\left(1+\frac{\left(1-p\right)f_{1}\left(x\right)}{pf_{0}\left(x\right)}\right)dx\;\\ =Cp\int_{-\infty }^{\infty }e^{-t^{2}/2}\ln\left(1+\frac{\left(1-p\right)}{p}e^{2(t-\alpha )\alpha }\right)dt,\left(t:=x+\alpha \right),\;\\ =Cp\int_{\left(-\infty ,\alpha )\cup [3\alpha ,\infty \right)}e^{-t^{2}/2}\ln\left(1+\frac{\left(1-p\right)}{p}e^{2(t-\alpha )\alpha }\right)dt\;\\ +Cp\int_{\alpha }^{3\alpha }e^{-t^{2}/2}\ln\left(1+\frac{\left(1-p\right)}{p}e^{2(t-\alpha )\alpha }\right)dt. \end{array}$$

Using the logarithmic inequality ln(1+x)<x for x>0, the first integral in Equation (A1) satisfies

(A2) $$\begin{array}{c} Cp\int_{\left(-\infty ,\alpha )\cup [3\alpha ,\infty \right)}e^{-t^{2}/2}\ln\left(1+\frac{\left(1-p\right)}{p}e^{2(t-\alpha )\alpha }\right)dt\;\\ <C\left(1-p\right)\int_{\left(-\infty ,\alpha )\cup [3\alpha ,\infty \right)}e^{-{\left(t-2\alpha \right)}^{2}/2}dt,\;\\ =2C\left(1-p\right)\int_{\alpha }^{\infty }e^{-u^{2}/2}du,\left(u:=t-2\alpha \right),\;\\ <2C\left(1-p\right)\int_{\alpha }^{\infty }ue^{-u^{2}/2}du,\;\left(∵u\geq \alpha >1\right),\;\\ =2C\left(1-p\right)e^{-\alpha^{2}/2}. \end{array}$$

Moreover, $0\leq 2\left(t-\alpha \right)\alpha \leq 4\alpha^{2}\;\mathrm{for}\;t\in \left[\alpha ,3\alpha \right)\;\mathrm{and}\;e^{4\alpha^{2}}>1$, for which using the last integral in Equation (A1) satisfies

(A3) $$\begin{array}{c} Cp\int_{\alpha }^{3\alpha }e^{-t^{2}/2}\ln\left(1+\frac{\left(1-p\right)}{p}e^{2(t-\alpha )\alpha }\right)dt,\;\\ \leq Cp\int_{\alpha }^{3\alpha }e^{-t^{2}/2}\ln\left(\frac{e^{4\alpha^{2}}}{p}\right)dt\;,\\ \leq Cp\ln\left(\frac{e^{4\alpha^{2}}}{p}\right)\int_{\alpha }^{\infty }e^{-t^{2}/2}dt\;,\\ \leq Cp\ln\left(\frac{e^{4\alpha^{2}}}{p}\right)e^{-\alpha^{2}/2}. \end{array}$$

The inequality in Equation (7) then follows by combining Equations (A2) and (A3) with (A1).

### Appendix A.2. Lower Bounds

We now derive a lower bound for the difference,

$$\begin{array}{c} \int_{-\infty }^{\infty }pf_{0}\left(x\right)\ln\left(pf_{0}\left(x\right)+\left(1-p\right)f_{1}\left(x\right)\right)dx-\int_{-\infty }^{\infty }pf_{0}\left(x\right)\ln\left(pf_{0}\left(x\right)\right)dx\\ =\int_{-\infty }^{\infty }p\frac{e^{-x^{2}/2}}{\sqrt{2\pi }}\ln\left(1+\frac{\left(1-p\right)}{p}e^{2(x-\alpha )\alpha }\right)dx. \end{array}$$

Notice that, by direct computation of second derivatives, it follows that the function $\varphi \left(t\right)=p\ln\left(1+\frac{1-p}{p}e^{2(t-\alpha )\alpha }\right)$ is a convex function. Thus, we can express

$$\begin{array}{c} \int_{-\infty }^{\infty }\frac{e^{-x^{2}/2}}{\sqrt{2\pi }}p\ln\left(1+\frac{\left(1-p\right)}{p}e^{2(x-\alpha )\alpha }\right)dx=E\left(\varphi \left(Z\right)\right), \end{array}$$

where *Z* is a standard Gaussian random variable. Applying Jensen’s inequality [27], we have

(A4) $$\begin{array}{cc} E(\varphi (Z)) & \geq \varphi (E(Z)),\;\\ & =p\ln(1+\frac{1-p}{p}e^{-2\alpha^{2}}). \end{array}$$

Similarly, one can show that

$$\begin{array}{c} \int_{-\infty }^{\infty }\left(1-p\right)f_{1}\left(x\right)\ln\left(pf_{0}\left(x\right)+\left(1-p\right)f_{1}\left(x\right)\right)dx-\int_{-\infty }^{\infty }\left(1-p\right)f_{1}\left(x\right)\ln\left(\left(1-p\right)f_{1}\left(x\right)\right)dx\\ =\int_{-\infty }^{\infty }\frac{e^{-x^{2}/2}}{\sqrt{2\pi }}\left(1-p\right)\ln\left(1+\frac{p}{1-p}e^{-2(x+\alpha )\alpha }\right)dx\\ =E(\Psi (Z)), \end{array}$$

where $\Psi \left(t\right)=\left(1-p\right)\ln\left(1+\frac{p}{1-p}e^{-2(t+\alpha )\alpha }\right)$ is a convex function. Using Jensen’s inequality,

(A5) $$\begin{array}{cc} E(\Psi (Z)) & \geq \Psi (E(Z))\;\\ & =\left(1-p\right)\ln\left(1+\frac{p}{1-p}e^{-2\alpha^{2}}\right). \end{array}$$
